# Supplementary material for: Nuclear IGF1R interact with PCNA to preserve DNA replication after DNA-damage in a variety of human cancers
Source: PLoS One. 2020 Jul 23;15(7):e0236291. doi: 10.1371/journal.pone.0236291 (PMC7377393; doi:10.1371/journal.pone.0236291)
Supplement: S1 Text — (DOCX) [file pone.0236291.s001.docx]

# Supplementary Material and Methods

## Immunohistochemistry

Immunohistochemistry (IHC) was carried out to control the specificity of the antibodies used in the Brightfield PLA experiments (described below) and to identify γ-H2AX foci. Formalin-fixed paraffin embedded (FFPE) samples were sectioned, manually deparaffinized and rehydrated. All antibodies were incubated overnight at 4°C. For anti-PCNA a highly proliferative cervical cancer and ductal mammary carcinoma (>95% positive tumor cells, negative stroma) and a low proliferating parathyroid adenoma (20% positive tumor cells) were used as controls. For anti-IGF1R we used formalin fixed paraffin block embedded *IGF1R* transfected R-cells (R+, see below) and non-transfected R-cells (R-) as controls. For anti-Rad18 we stained lymphatic germinal centers (lymphocytes were negative). All IHC evaluation and scoring was carried out by a clinical pathologist. Representative photomicrographs of IGF1R antibodies are shown in Supplementary Figure 1.

## Immunofluorescence

To visualize POLH foci, cover slides with treated HeLa cells attaching were fixed with 4% paraformaldehyde in PBS at RT for 15 mins and permeabilized with 0.1% triton X-100 in PBS at RT for 10 mins. The slides were then blocked with 5% BSA, 5% donkey serum, 0.3% Triton X-100 in PBS for 30min at 37 °C followed by immunostaining of primary antibody (1:100 anti-POLH, Abcam ab186677) and 1:200 Alexa Fluor 594 conjugated secondary antibody (ThermoFisher A-11012). The slides were then mounted with VECTASHIELD® Vibrance™ Antifade Mounting Medium with DAPI as nuclear counterstaining reagent. Photographs of random areas in each condition were captured by Zeiss Zxio imager M2 microscopy and POLH foci number of at lease 40 cells were counted. The foci per cell were calculated in each experiment conditions and statistical analyses were performed with Microsoft Excel. *P*-value< 0.05 was considered significant.

## *In situ* Proximity Ligation Assay (PLA)

Cells were seeded on coverslips and fixed with 4% PBS buffered paraformaldehyde, permeabilized by 0.1% Triton X-100. After blocked for 30 min in blocking buffer (5% BSA, 5% donkey serum, 0.3% Triton X-100 in PBS), the staining was implemented following the manufactory’s instruction (Duolink® In Situ Detection Reagents, Sigma-Aldrich). Interaction were detected using Zeiss LSM710 confocal microscope and analysed using Image J software. To perform the PLA on tissue sections, the slides were re-hydrated by incubation in Xylene for 10 min, different ethanol solutions (3x 99 %, 2x 95 % and 70 %) for 2min each and running water for 2 min. For Antigen retrieval the sections were incubated in citrate antigen retrieval solution (S1699 Dako, United States) and microwaved at 750 W for 8 min and then at 350 W for 20 min (sub-boiling). After cooling the tissue sections in a water bath with running water for 10 min, the intrinsic peroxidase activity was quenched by incubation in a H_2_O_2_ solution (1:60) (Sigma-Aldrich, Sweden AB) and incubated in the dark at room temperature for 30 min. The following steps were performed according to the Duolink^®^ PLA Brightfield Protocol (Sigma-Aldrich). The antibodies used in PLA were diluted in Antibody Diluent as following: for IGF-1R/PCNA colocalization: 1:10 mouse anti-IGF1R (sc-390130, Santa Cruz) and 1:150 rabbit anti-PCNA (HPA030522, Sigma); for RAD18/PCNA colocalization: 1:500 mouse anti-Rad18 (WH0056852M1, Sigma) and 1:200 rabbit anti-PCNA (HPA030522, Sigma); for IGF-1R/RAD18 colocalization: and 1:500 rabbit anti-Rad18 (WH0056852M1, Sigma) 1:200 mouse anti-IGF1R (9750, Cell Signalling Technology).

A group (n = 15) of the clinical cases evaluated by *in situ* PLA was stained twice to verify the reproducibility between staining batches.

## Cell lines and reagents

R- (MEF *igf1r-/-*) and R+ (R- overexpressing IGF1R) were from Dr. R. Baserga (Thomas Jefferson University, USA). Melanoma cell line A375 were from Dr. L. Girnita (Karolinska Institutet, Sweden). Colorectal cancer cell lines DLD1 and HCT116, prostate cancer cell line DU145, cervical cancer cell line HeLa, ovarian cancer cell line OVCAR-3 and TOV112D, osteosarcoma cell line U2OS, synovial sarcoma cell line CME1, breast cancer cell line MCF7, lung cancer cell line H1299, Ewing sarcoma cell line RD-ES were purchased from American Type Culture Collection (ATCC). All cell lines were cultured in DMEM high glucose (41965039, Thermofisher Scientific) with 10% Fetal Bovine Serum (16000044, Thermofisher Scientific), with the exception of OVCAR-3 being cultured in RPMI-1640 (21875091, Thermofisher Scientific) with 10% FBS and Insulin-Transferrin-Selenium (41400045, Thermofisher Scientific). All cell lines were maintained at 37 °C in a humidified atmosphere containing 5% CO2 Mycoalert™ and checked for mycoplasma using kit (Lonza, Switzerland). All human cell lines were short tandem repeat-authenticated using AmpFLSTR® Identifier® plus kit (Applied Biosystems). IGF-1R tyrosine kinase inhibitor NVP-AEW541 (NVP), human insulin-like growth factor-1 (IGF-1), dimethyl sulfoxide (DMSO), hydroxurea (HU), N-Ethylmaleimide (NEM), β-mercaptoethanol were all purchased from Sigma.

## Immunoblotting and immunoprecipitation

Cells were lysed in modified RIPA buffer (50mM Tris, pH 7.4, 150 mM NaCl, 1% Nonidet P-40, 1 mM EDTA, 0.25% sodium deoxycholate) containing Protease and Phosphatase Inhibitor Cocktail (78447, Thermofisher Scientific). Primary antibodies used in this research included the following: rabbit anti-IGF1R (3024, Cell Signaling Technology), mouse anti-PCNA (2586, Cell Signaling Technology), mouse anti-β-Actin (A5441, Sigma), rabbit anti-ub-PCNA (13439, Cell Signaling Technology) and mouse anti-Rad18 (WH0056852M1, Sigma). Membranes were incubated with secondary anti-rabbit/mouse/goat IgG horseradish peroxidase-conjugated antibodies (NA934 and NA931 from GE Healthcare and 31402 from Pierce) followed by signal detection using enhanced luminescence Hyperfilm ECL (GE Healthcare).

Rabbit anti-PCNA (ab18197, Abcam) and rabbit anti-IgG (sc-66931, Santa Cruz) were cross-linked to magnetic protein G Dynabeads using Dynabeads® antibody coupling kit (14311D, Invitrogen). 2–4 mg of cell lysates were incubated with cross-linked antibody at concentrations of 4–7µg of antibody/mg beads overnight at 4 °C. The immune complexes were washed three times with lysis buffer and eluted by boiling in SDS sample buffer (NP0007, Invitrogen). Subsequent immunoblottings were performed as described above.
