# Supplementary figures and images for: Nuclear IGF1R interact with PCNA to preserve DNA replication after DNA-damage in a variety of human cancers
Source: PLoS One. 2020 Jul 23;15(7):e0236291. doi: 10.1371/journal.pone.0236291 (PMC7377393; doi:10.1371/journal.pone.0236291)

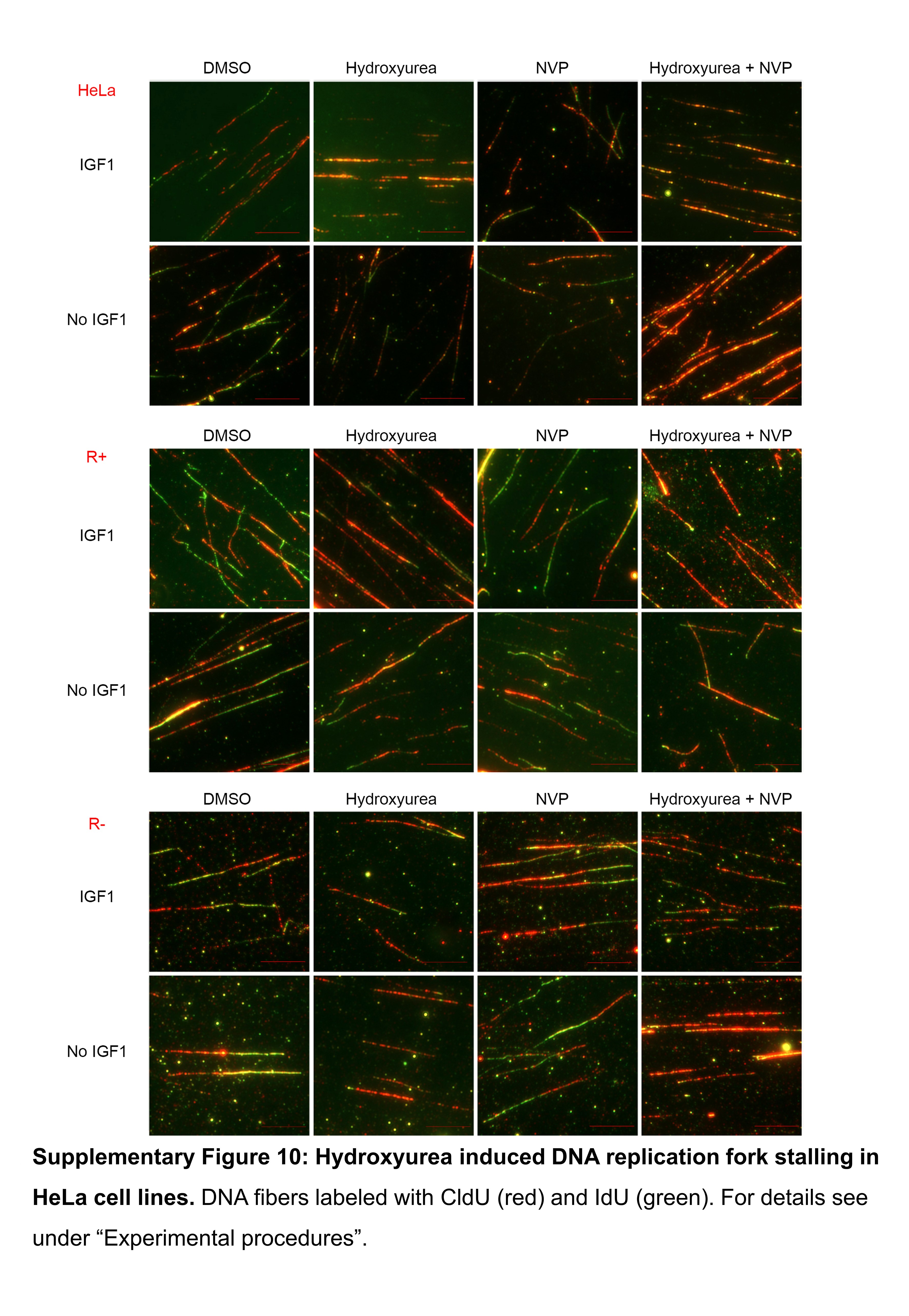

Supplement: S10 Fig — (JPG) [file pone.0236291.s011.jpg]

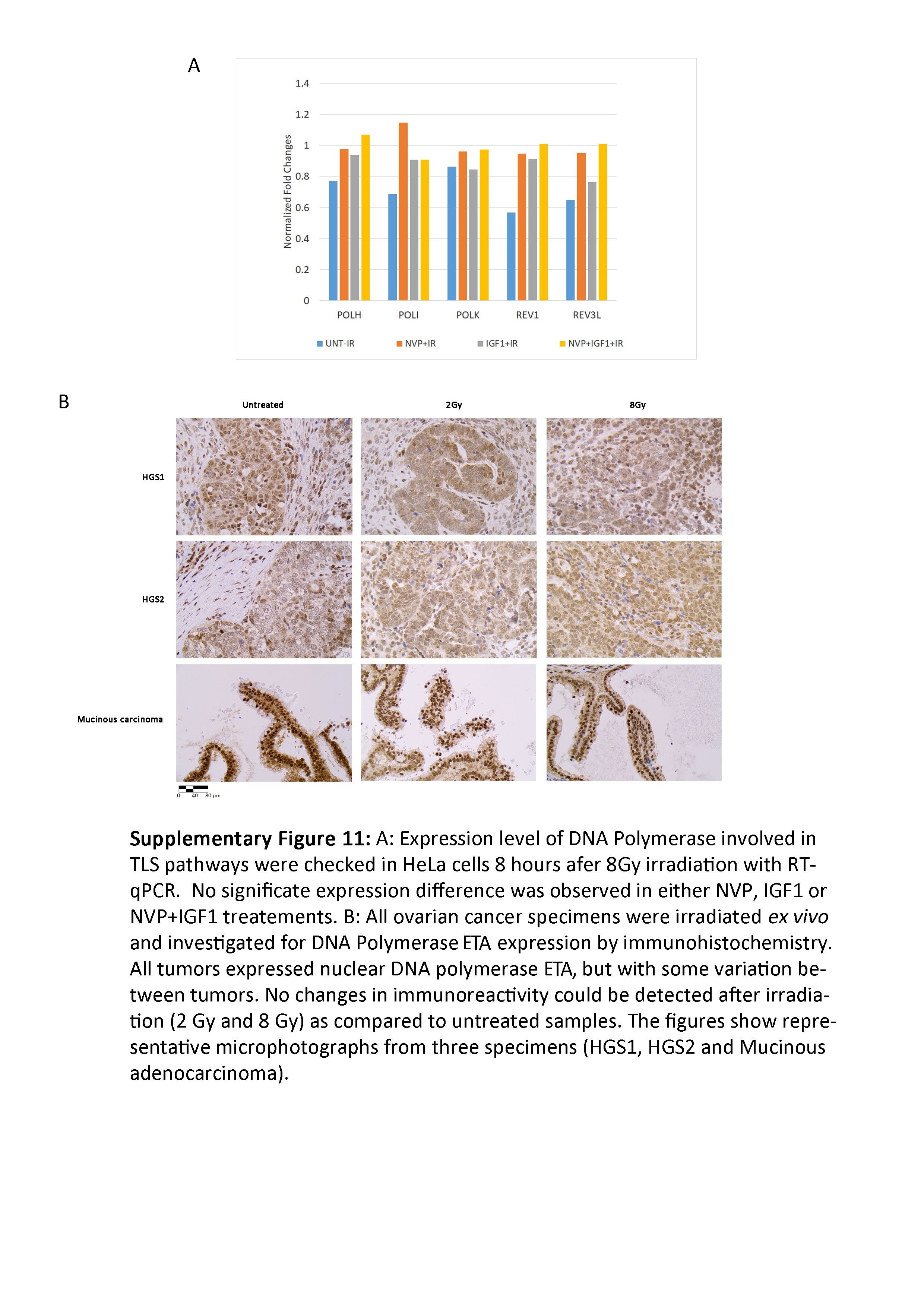

Supplement: S11 Fig — (JPG) [file pone.0236291.s012.jpg]

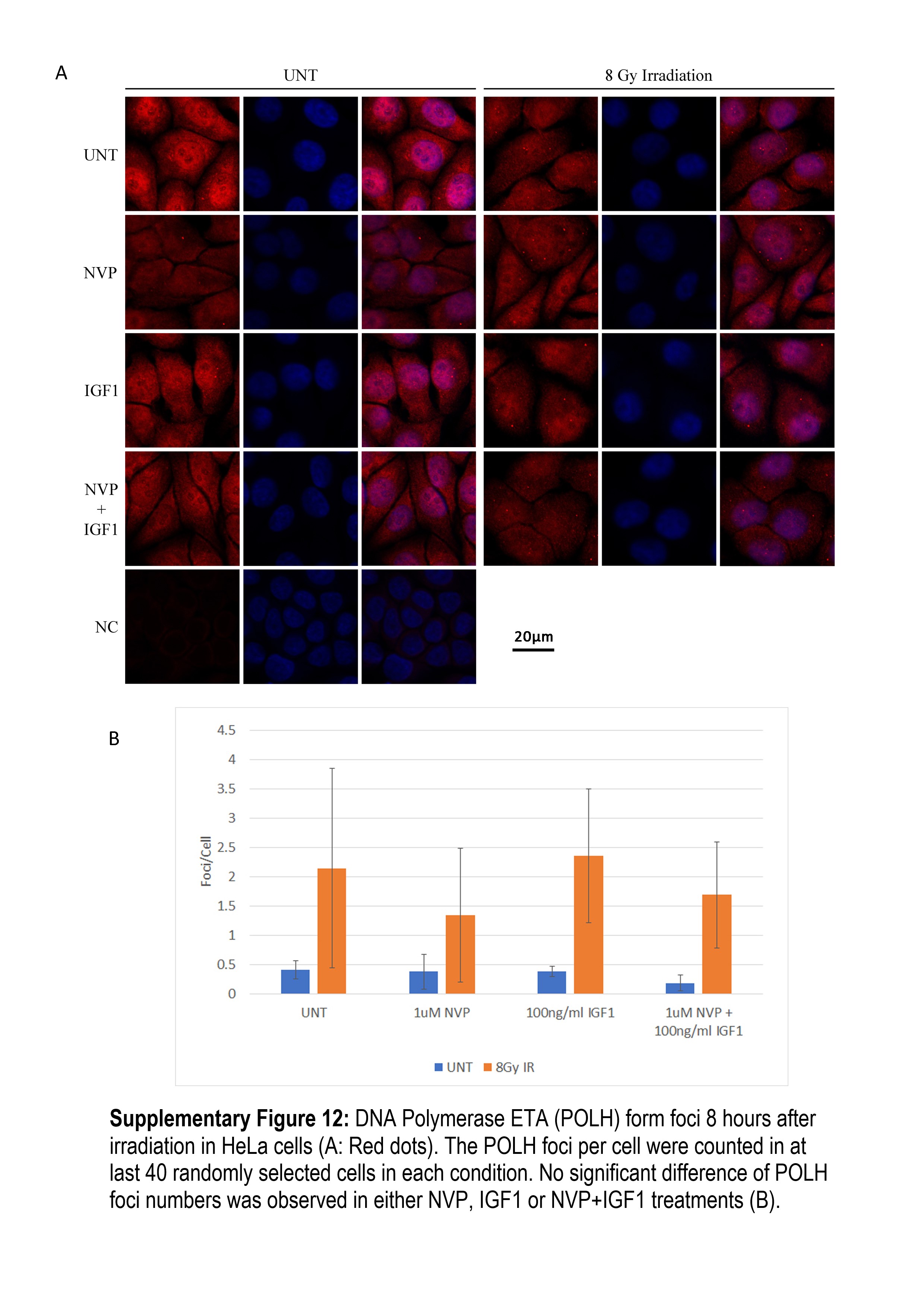

Supplement: S12 Fig — (JPG) [file pone.0236291.s013.jpg]
